# Editorial Comment to Castration‐resistant prostate cancer diagnosed during leuprorelin treatment for spinal and bulbar muscular atrophy

**DOI:** 10.1002/iju5.12465

**Published:** 2022-05-10

**Authors:** Maria Pennuto, Monica Montopoli, Carlo Rinaldi

**Affiliations:** ^1^ Department of Biomedical Sciences University of Padova Italy; ^2^ Veneto Institute of Molecular Medicine (VIMM) Padova Italy; ^3^ Department of Pharmaceutical and Pharmacological Sciences University of Padova Padova Italy; ^4^ Institute of Oncology Research Oncology Institute of Southern Switzerland Bellinzona Switzerland; ^5^ Department of Paediatrics University of Oxford UK

Androgen receptor (AR) is the main mediator of androgen signaling. Androgens are sex steroid hormones released into the bloodstream in response to activation of the hypothalamic–pituitary‐target endocrine gland axis. Free androgens enter the cells and bind to AR in the cytosol. In response to ligand binding, AR shuttles to the nucleus and works as a ligand‐activated transcription factor. AR is widely expressed, from reproductive organs to skeletal muscle and brain. Hundreds of AR mutations have been linked to androgen insensitivity syndrome and prostate cancer. The expansion of a microsatellite CAG tandem repeat is the only *AR* mutation linked to neurodegeneration. SBMA symptoms fully manifest in males due to high circulating androgen levels. Androgen deprivation therapy (ADT) has shown promise in SBMA patients.^1^, ^2^ ADT however is associated with side effects, spanning from muscle atrophy to sexual dysfunction and depression. ADT can also suppress hormone‐naïve prostate cancer until a status of castration resistance is selectively established by mechanisms not entirely understood, comprising AR point mutations or overexpression, altered androgen biosynthesis, and constitutively active AR splice variants.

The case report from Atsushi *et al*. reignites a long‐standing debate whether the size of the CAG microsatellite expansion in the *AR* gene influences development and progression of prostate cancer, the second most prevalent malignancy in men worldwide.^3^ It has been observed that the size of the CAG repeats inversely correlates with the AR transcriptional activity and the risk of developing prostate cancer,^4^ therefore suggesting that SBMA patients are less at risk compared to the general population.

With only a handful of reported cases and lack of solid epidemiological data, this question remains open. Additional mechanisms that may drive cancer initiation and progression beyond AR include age and loss of DNA repair, which are both a feature of SBMA and other polyglutamine disease. Those may be responsible for the insurgence of androgen‐independent and more clinically aggressive prostate cancers in SBMA patients.^5^ As long‐term ADT is currently in clinical use in Japan for patients with SBMA, the links between microsatellite expansion in AR, androgen deprivation, and prostate cancer requires investigation to optimally balance benefits and harms of such treatment strategies for patients.

## Conflict of interest

The authors have no conflict of interest to report.

## Author contributions

Maria Pennuto: Conceptualization; writing – original draft. Monica Montopoli: Conceptualization; writing – original draft. Carlo Rinaldi: Conceptualization; writing – original draft.
